# Convergence of independent *DISC1* mutations on impaired neurite growth via decreased *UNC5D* expression

**DOI:** 10.1038/s41398-018-0281-9

**Published:** 2018-11-08

**Authors:** Priya Srikanth, Valentina N. Lagomarsino, Richard V. Pearse, Meichen Liao, Sulagna Ghosh, Ralda Nehme, Nicholas Seyfried, Kevin Eggan, Tracy L. Young-Pearse

**Affiliations:** 1 Ann Romney Center for Neurologic Diseases, Brigham and Women’s Hospital and Harvard Medical School, Boston, MA USA; 2 Harvard Stem Cell Institute, Department of Stem Cell and Regenerative Biology, Harvard University, Cambridge, MA 02138 USA; 3 Stanley Center for Psychiatric Research, Broad Institute of MIT and Harvard, Cambridge, MA 02142 USA; 4 Department of Biochemistry, Department of Neurology, Emory University School of Medicine, Atlanta, GA 30322 USA

## Abstract

The identification of convergent phenotypes in different models of psychiatric illness highlights robust phenotypes that are more likely to be implicated in disease pathophysiology. Here, we utilize human iPSCs harboring distinct mutations in *DISC1* that have been found in families with major mental illness. One mutation was engineered to mimic the consequences on DISC1 protein of a balanced translocation linked to mental illness in a Scottish pedigree; the other mutation was identified in an American pedigree with a high incidence of mental illness. Directed differentiation of these iPSCs using *NGN2* expression shows rapid conversion to a homogenous population of mature excitatory neurons. Both *DISC1* mutations result in reduced DISC1 protein expression, and show subtle effects on certain presynaptic proteins. In addition, RNA sequencing and qPCR showed decreased expression of *UNC5D, DPP10, PCDHA6*, and *ZNF506* in neurons with both *DISC1* mutations. Longitudinal analysis of neurite outgrowth revealed decreased neurite outgrowth in neurons with each *DISC1* mutation, which was mimicked by *UNC5D* knockdown and rescued by transient upregulation of endogenous *UNC5D*. This study shows a narrow range of convergent phenotypes of two mutations found in families with major mental illness, and implicates dysregulated netrin signaling in DISC1 biology.

## Introduction

One approach to the study of major mental illness is to study disease-associated mutations in cell and animal model systems. However, such mutations often result in relatively subtle alterations in neuronal structure, function, and connectivity. The study of multiple mutations linked to major mental illness in parallel enables the identification of shared phenotypes across disease-associated mutations. This allows for discernment of those phenotypes that are unique to a specific disease-associated mutation from those that may be common to the disease process. Identification of convergent phenotypes will therefore further elucidate disease pathophysiology and hopefully facilitate development of interventions that may alter the course of the disease.

*DISC1* has been implicated in major mental illness by rare mutations that have been linked to neuropsychiatric disease, including schizophrenia, bipolar disorder, and autism spectrum disorder^1–4^. Controversy exists surrounding the relevance of *DISC1* mutation to psychiatric disease given the lack of *DISC1* association in large GWAS studies, and the limited number of pedigrees with *DISC1* disruption and mental illness (two described to date with more than one carrier). DISC1 was first identified in a Scottish pedigree in which a *t*(1;11) translocation interrupting *DISC1* co-segregates with major mental illness^2^. One concern with this pedigree is that the translocation does not perfectly segregate with the disease, and other aspects of the genetic background may be contributing to risk. Subsequent studies of this family have shown an association of the balanced translocation with a reduction in cortical thickness, a phenotype which was shared with subjects with schizophrenia^2,5,6^. The second pedigree is from a smaller American family that harbors a 4 bp deletion at the C-terminus of the gene^3^. Studies of *DISC1* to date implicate the gene as a rare variant that predisposes to disease in select individuals, which GWAS studies are not adept in identifying^7^. This principle is demonstrated in Alzheimer disease by the strong pathophysiological insights gained from rare mutations in *APP, PSEN1*, and *PSEN2* despite the absence of linkage of these genes to disease in GWAS analyses^8^. Given these examples and the extensive data linking *DISC1* variants to defects in neurodevelopmental processes, we believe that the study of rare disease-associated *DISC1* mutations provides an opportunity to elucidate disease-relevant phenotypes important to the proper development of the human brain. In spite of the controversy over *DISC1* genetic findings, there is consensus that DISC1 has an important role in neurodevelopment^1^.

Two different *DISC1* mutations have been studied in human induced pluripotent stem cell (iPSC)-derived neurons. One, developed by our group, is modeled after the balanced *t*(1;11) translocation described above that interrupts the coding sequence of *DISC1* early in exon ^2,9,10^. We used TALENs to create isogenic iPSC lines with a frameshift mutation in exon 8 of *DISC1*, which induced a nearby premature stop codon, thereby closely recapitulating the effects of *DISC1* interruption at the site of the balanced translocation^11^. Another mutation is a 4 bp deletion at the end of DISC1 exon 12, which was identified in a proband with schizophrenia in an American pedigree^3,12^. In the Song and Ming labs, iPSCs were generated from family members of this pedigree and were additionally genetically modified to either introduce or correct the DISC1 mutation of interest^12^. Both our group and the Song/Ming groups employed an embryoid aggregate-based differentiation protocol in our published studies, with some differences in the specific methods across studies. While the embryoid aggregate-based protocol provides a highly valuable technique for studying neurodevelopment, this differentiation method results in a heterogeneous pool of neural cells that follows a protracted human developmental timeline^13^. This can complicate analyses requiring a homogenous cell population of mature neurons, which may not develop in these cultures for several months.

The advancement of lineage reprogramming has yielded differentiation protocols with accelerated timescales and, depending on the protocol, relatively homogenous cell fates. These methods employ forced expression of critical regulators of cell fate, driving differentiation toward-specific cell types. In comparison to their embryoid-aggregate-based counterparts, transdifferentiation protocols often result in cells that mature more quickly than aggregate-derived cells, and that overcome the protracted development and differentiation of aggregate-based methods. While protocols that faithfully recapitulate development offer insights into disease-associated neurodevelopmental defects, lineage reprogramming provides an opportunity to examine a homogenous population of mature neurons. Increased culture homogeneity also reduces line-to-line differentiation variability, which may reveal otherwise-obscured, subtle, or cell-type-specific effects of disease mutations. Lineage reprogramming thus allows an investigator to bypass neurodevelopmental phenotypes to study the effects of a given mutation in a relatively mature neuron.

Multiple protocols exist for generating glutamatergic neurons from other cell types^14–21^. Prior studies have used expression of *ASCL1, POU3F2, and MYTL* to generate mature neurons from cells containing autism and psychiatric disease risk variants^22,23^. Another method utilizes Neurogenin-2 (*NGN2*) expression, which directly converts iPSCs to cerebral cortical layer 2/3 excitatory neurons^14^, and has been used to study disease-associated mutations in models of autism and schizophrenia^24–26^, tuberous sclerosis^27^, epileptic encephalopathy^28^, and neurodegenerative diseases^29,30^.

We previously showed that *DISC1* exon 8 disruption alters WNT activity and neural progenitor and neuronal cell fate based on gene and protein expression^11^, without global effects of altered pre- or post-synaptic gene or protein expression. The study of the *DISC1* 4 bp deletion found that *DISC1* mutation led to altered expression of presynaptic proteins, thereby reducing neuronal activity^12^. We sought to directly compare these *DISC1* mutation models using the same differentiation protocol, in order to determine the convergent phenotypes that exist with both mutations and to better understand altered biology across these models. Given the synaptic phenotype found with *DISC1* 4 bp deletion, we aimed to compare phenotypes of *DISC1* mutation in mature, excitatory neurons using NGN2 transduction. This protocol both enhances consistency due to the homogenous cell population generated^14^, and aids in the elimination of confounding effects of the developmental phenotype observed with *DISC1* exon 8 mutation, which could potentially mask a later synaptic phenotype through alteration of cell fates.

We therefore utilized iPSCs with *DISC1* exon 8 mutation (with isogenic wild-type lines) and with the *DISC1* exon 12 4 bp deletion (with wild-type lines derived from family members, kindly provided by Drs. Song and Ming) to generate mature glutamatergic neurons using NGN2 transduction and compare neuronal characteristics. In neurons, neither *DISC1* mutation resulted in altered global expression of synaptic proteins, but did show subtle defects in a subset of presynaptic proteins. Using RNAseq to evaluate the transcriptome, we found convergence of both *DISC1* mutations on significantly decreased expression of just 4 genes after controlling for multiple comparisons, including *UNC5D*, a netrin receptor. Analysis of neurite outgrowth showed a persistent decrease in neurite length with both exon 8 and exon 12 *DISC1* mutations. This phenotype was induced in wild-type neurons by *UNC5D* knockdown and rescued in *DISC1* mutant neurons by *UNC5D* upregulation. Thus, two independent *DISC1* mutations linked to major mental illness result in impaired neurite outgrowth via decreased expression of *UNC5D*, implicating this pathway in the pathogenesis of psychiatric disorders.

## Materials and methods

### Induced neuron differentiation

Induced neurons were generated as described^14^, with minor modifications described below. iPSCs were plated in mTeSR1 media at a density of 95K cells/cm^2^ on Matrigel-coated plates for viral transduction. Viral plasmids were obtained from Addgene (plasmids #19780, 52047, 30130). Lentiviruses were obtained from Alstem with ultrahigh titers and used at the following concentrations: pTet-O-NGN2-puro: 0.1 µl/50K cells; Tet-O-FUW-eGFP: 0.05 µl/50K cells; Fudelta GW-rtTA: 0.11 µl/50K cells. Transduced cells were dissociated with Accutase and plated onto Matrigel-coated plates at 50,000 cells/cm^2^ in mTeSR1 (day 0). On day 1, media were changed to KSR media with doxycycline (2 µg/ml, Sigma). Doxycyline was maintained in the media for the remainder of the differentiation. On day 2, media were changed to 1:1 KSR: N2B media with puromycin (5 µg/ml, Gibco). Puromycin was maintained in the media throughout the differentiation. On day 3, media were changed to N2B media + 1:100 B27 supplement (Life Technologies), and puromycin (10 µg/ml). From day 4 on, cells were cultured in NBM media + 1:50 B27 + BDNF, GDNF, CNTF (10 ng/ml, Peprotech).

### Embryoid aggregate differentiation

Neuronal differentiation via embryoid aggregate method was performed as previously described^31^. Briefly, iPSC colonies were removed from MEFs and cultured as embryoid aggregates in suspension for 4 days in iPSC media (without FGF2), followed by 2 days in N2 neural induction media. Day 7 aggregates were plated onto Matrigel-coated 6-well plates and maintained in N2 neural induction media, forming neuroepithelial structures. At day 17, neural rosettes were enzymatically isolated using STEMDiff Neural Rosette Selection Reagent (Stemcell Technologies) and cultured in suspension for 7 days in N2/B27 neural induction media containing cAMP (1 µM, Sigma) and IGF-1 (10 ng/ml, Peprotech). Day 24 neural aggregates were dissociated using Accutase in the presence of 10 µM ROCK inhibitor and plated for final differentiation in neural differentiation media containing cAMP (10 µM, Sigma), IGF-1, BDNF, and GDNF (10 ng/ml, Peprotech).

### Induced neuron protocol

- KSR media: Knockout DMEM, 15% KOSR, 1× MEM-NEAA, 55 µM beta-mercaptoethanol, 1× GlutaMAX (Life Technologies).
- N2B media: DMEM/F12, 1× GlutaMAX (Life Technologies), 1× N2 supplement B (Stemcell Technologies), 0.3% dextrose (d-(+)-glucose, Sigma).
- NBM media: Neurobasal medium, 0.5× MEM-NEAA, 1× GlutaMAX (Life Technologies), 0.3% dextrose (d-(+)-glucose, Sigma).

### Embryoid aggregate protocol

- MEF media: DMEM, 10% FBS, 100 U/ml Penicillin-Streptomycin, 2 mM l-glutamine (Life Technologies).
- iPSC media: DMEM/F12, 20% KOSR, 1× MEM-NEAA, 1× Penicillin-Streptomycin-Glutamine, 55 µM beta- mercaptoethanol (Life Technologies).
- N2 neural induction media: DMEM/F12, 1× N2 supplement, 1× MEM-NEAA (Life Technologies), 2 µg/ml heparin (Sigma).
- N2/B27 neural induction media: DMEM/F12, 1× N2 supplement, 1× B27 supplement, 1× MEM-NEAA (Life Technologies), 2 µg/ml heparin (Sigma).
- Neural differentiation media: Neurobasal medium, 1× N2 supplement, 1× B27 supplement, 1× MEM-NEAA (Life Technologies), 2 µg/ml heparin (Sigma).

### RNA sequencing

Double-stranded cDNA was synthesized using the SuperScript III reverse transcriptase protocol with random hexamers on 1 ng mRNA from each sample analyzed. Sequencing libraries were generated by processing the double-stranded cDNA product through the Illumina Nextera tagmentation library protocol. Multiplexed libraries were sequenced on an Illumina NextSeq 500 to a depth of 433 million paired-end reads (75 bases per read) total. RNAseq reads were quality trimmed, then quantified using the Kallisto pseudoalignment quantification program (58) (v0.42.4) running 100 bootstraps against a kallisto index generated from ChGR38 with a k-mer length of 31. Kallisto quantified samples are compared using Sleuth (59) (v0.28.1) in R Studio (v3.4.0 of R; v1.0.143 of R Studio). The ComBat algorithm was used to remove variance induced by differentiation round^32^.

### Single-cell qRT-PCR

For single cell experiments, the NGN2 protocol was modified to include dual SMAD inhibition and WNT inhibition during a critical window of differentiation^33^. Day 4-induced neurons were co-cultured with mouse astrocytes. WT^ex8^ lines A & B and MUT^ex8^ lines A & D were used for this single-cell analysis. Day 28 cultures were single-cell sorted for GFP expression (marking transduced neurons) into lysis buffer (10 mM Tris, pH 8.0, 0.1 mM EDTA with 0.5% NP40 [Thermo Scientific PI-28324] and 100 U/ml SUPERase•In™ [Ambion AM2696]). Sorted cells in 96-well plates were flash-frozen on dry ice and kept at −80 °C until further processing. Single-cell gene expression profiling was performed using the Fluidigm Biomark dynamic array according to the manufacturer’s protocol. Quantitative RT-PCR was performed using TaqMan Gene Expression Assays (Applied Biosystems) on the Biomark 96.96 Dynamic Array system (Fluidigm).

### qRT-PCR

RNA was extracted using the Pure Link RNA Mini Kit (Life Technologies) and reverse transcribed using SuperScript II (Life Technologies). cDNA was used for qPCR with Fast SYBR Green Master Mix (Life Technologies) on a ViiA 7 System (Life Technologies). Samples were run using at least 3 technical replicates (*n* in figures represent biological replicates only). Data were normalized to GAPDH expression using the ΔΔC_T_ method as previously described^34^. Primers are listed in Table 1.

### Immunocytochemistry and microscopy

Cells were fixed with 4% paraformaldehyde (Sigma), followed by membrane permeabilization and blocking with 0.2% Triton X-100 (Sigma) and 2% donkey serum (Jackson Immunoresearch) in PBS for 1 h at room temperature. Cells were then incubated with primary antibodies overnight at 4 °C, secondary antibodies for 1 h at room temperature, and 1:1000 DAPI (Life Technologies) for 10 min, with multiple washes between each step. Antibodies used are listed in Table 2. Cells were stained for F-actin as indicated, using 670 Fluorescent Phalloidin (Cytoskeleon, Inc., PHDN1) following protocol from manufacturer. Samples were imaged using Zeiss LSM710 or LSM880 confocal microscopes and Zen black software. Zen black and FIJI were used to pseudo-color images and add scale bars.

### Western blots

Lysates were prepared in a buffer containing 1% NP40, 10 mM EDTA, 150 mM NaCl, 50 mM Tris, cOmplete Protease Inhibitors and phosSTOP (Roche). BCA protein assays were performed on all samples to normalize for protein content (Pierce). Equal protein amounts were loaded onto 4–12% Bis-Tris NuPAGE gels (Life Technologies) and transferred to nitrocellulose membranes. Blots were either incubated with HRP-conjugated secondary antibodies and developed using ECL substrate (Pierce) or incubated with fluorophore-conjugated secondary antibodies and imaged on the Odyssey system (LI-COR).

### LC–MS/MS proteomics

Day 21 iNs were lysed in urea lysis buffer (8 M urea, 100 mM NaHPO_4_, pH 8.5), including 5 µl (100× stock) HALT protease and phosphatase inhibitor cocktail (Thermo Fisher), and used for label-free LC–MS/MS proteomics through the Emory School of Medicine Proteomics Core, as described^35^. The ComBat algorithm was used to remove variance induced by differentiation round^32^.

### Neurite outgrowth assay

Day 4 iNs were dissociated and plated on Matrigel coated 96-well clear plates (Greiner Bio-One, 655090) at ~15,000 cells/cm^2^. Plates were cultured and imaged in an Essen live cell analysis IncuCyte system for up to 120 h (DIV 9). Neurite outgrowth analysis was performed using IncuCyte ZOOM software.

### *UNC5D* knockdown and overexpression

*UNC5D knockdown:* Day 4 iNs were dissociated and plated in Matrigel coated 96-well µclear plates (Greiner Bio-One, 655090) at 78,125 cells/cm^2^. Approximately 12 h later, iNs were transduced with either vehicle, empty lentivirus, or 4 lentiviruses encoding UNC5D shRNA (Sigma: TRCN0000419223, TRCN0000061318, TRCN0000438007, TRCN0000061322). Twelve hours after infection, lentivirus was removed and fresh D4 media were added to all wells. Cells were cultured in the IncuCyte for 120 h before fixation with 4% paraformaldehyde (Sigma) or lysing for RNA analysis.

*UNC5D activation:* UNC5D activation studies using CRISPR/Cas were designed based upon concepts presented in two studies^36,37^. A dCas9-VPR plasmid (Addgene #63798^38^) was co-transfected with red fluorescent protein (RFP) and gRNAs targeted to the promoter of UNC5D: #1 CGAGCGGGGGGGGGAGCTGC, #2 GAGGCCGCTCCACGTGCCCC, #3 GGCGGGGCCCGAAGCGCCCC. NGN2-transduced iPSCs were plated onto Matrigel-coated plates in mTeSR1 (day 0). On D2 of iN differentiation, cells were transfected with RFP, gRNAs, and dCas9-VPR following the Lipofectamine 2000 protocol (Thermo Fisher Scientific). On D4, iNs were dissociated and plated on Matrigel coated 96-well µclear plates (Greiner Bio-One, 655090) at ~75,000 cells/cm^2^. Plates were cultured and imaged in the Essen live cell analysis IncuCyte system for up until day 12. Due to low transfection efficiency, neurite outgrowth analysis was performed using IncuCyte ZOOM software on RFP positive neurites only.

### Data collection and statistics

Data were analyzed using GraphPad PRISM 7 software. Values are expressed as means ± SEM. Statistical significance was tested as indicated in figure legends.

## Results

### *DISC1* mutation does not significantly alter cell fate or electrophysiological activity in NGN2 induced neurons

**Fig. 1:**
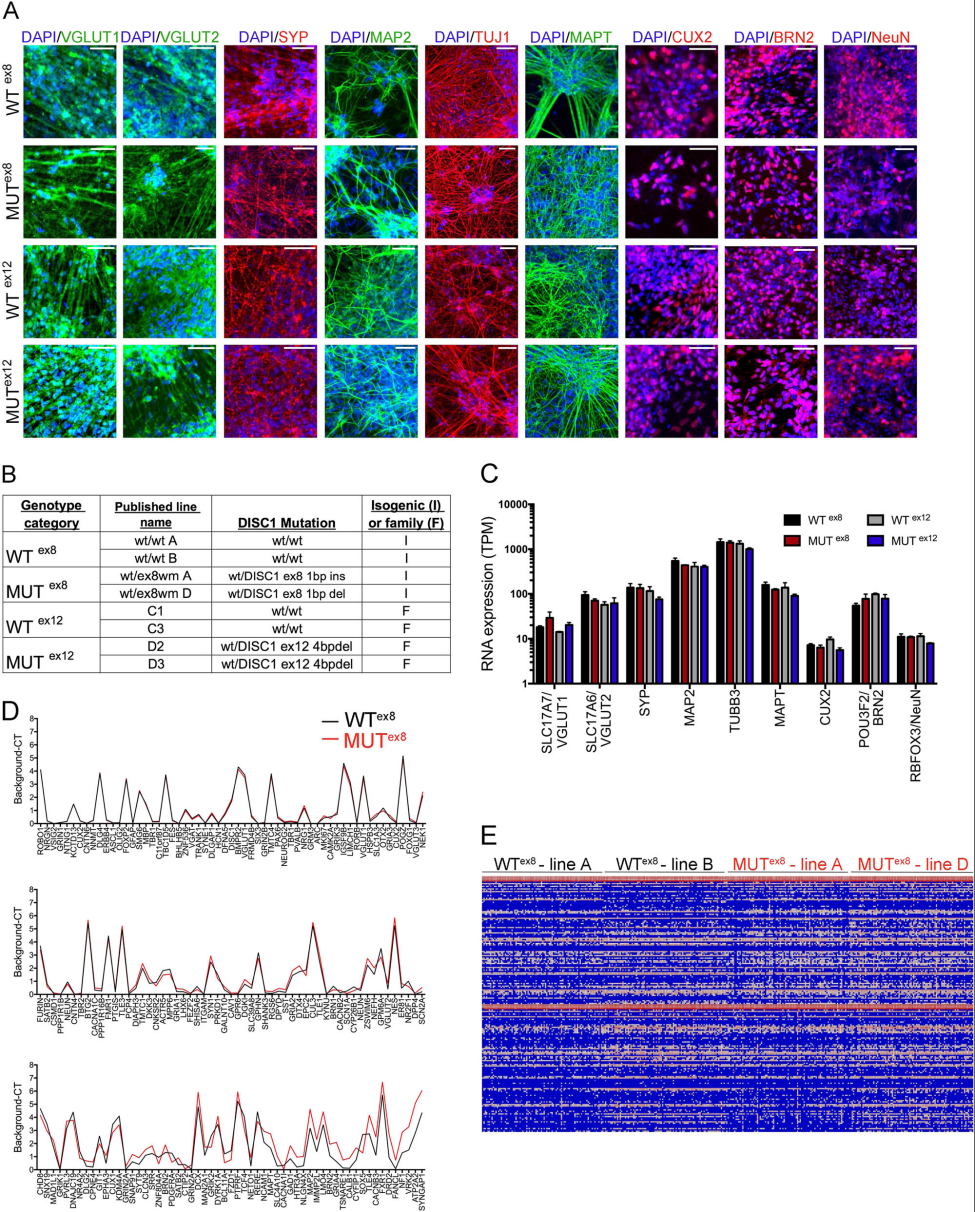
NGN2 transduction efficiently and consistently converts iPSCs to mature excitatory neurons across iPSC lines. **a** iPSCs were infected with NGN2 and cultured as iNs to day 21, and immunostained for neuronal markers as shown. Scale bars = 50 μm. **b** Table summarizing lines used, including genotype as referred to here, lines as named in prior papers^11,12^, *DISC1* genotype, and type of control. **c** RNA sequencing was performed on day 21 iNs; TPM is shown for the same neuronal markers shown in **a**. **d** Single-cell multiplex qRT-PCR (Fluidigm) of 184 cells each from WT^ex8^ and MUT^ex8^ lines for 187 genes. *Y*-axis shows background-C_T_, with higher numbers representing higher expression. Genes are arranged left to right and top to bottom from most similar expression across WT^ex8^ and MUT^ex8^ to most divergent expression. **e** Heatmap of single-cell data in D along with housekeeping genes, grouped by line and genotype. Columns single cells, rows single genes

Glutamatergic neurons were generated using viral *NGN2* transduction^14^. Immunostaining of day 21 cells confirmed that *NGN2* expression induced rapid neuronal fate conversion and resulted in induced neurons (iNs) expressing excitatory synaptic markers VGLUT1 and VGLUT2, the presynaptic marker SYP, neuronal cytoskeletal proteins MAP2, TUJ1, and TAU (MAPT), the mature neuronal marker NEUN, and cortical upper-layer neuronal transcriptional proteins CUX2 and BRN2 (Fig. 1a). Isogenic wild type and *DISC1* exon 8 mutant lines (WT^ex8^ and MUT^ex8^) and familial wild type and *DISC1* exon 12 4 bp deletion lines (WT^ex12^ and MUT^ex12^) were all similarly differentiated (Fig. 1a). Two independent lines of each genotype were used, with nomenclature corresponding to prior publications shown in Fig. 1b^11,12^. Comparison of gene expression in iNs versus embryoid aggregate-differentiated neurons revealed expected decreases in expression of neural progenitor markers and increased expression of mature neuronal and excitatory synaptic markers (Supp Fig. 1). RNA sequencing was performed on day 21 cultures; RNA expression of the genes corresponding to immunostaining markers in Fig. 1a is shown in Fig. 1c. There were no significant differences in expression of these neuronal markers across the 4 genotypes studied. WT^ex8^ and MUT^ex8^ lines additionally were used for single-cell multiplex qRT-PCR with the Fluidigm platform, analyzing expression of 187 genes relevant to neuronal differentiation and psychiatric disease (Supp. Table 1). At a single cell level, expression of the majorinterrupts the coding sequenceity of these genes was very consistent across individual cells, reinforcing the efficiency and consistency of the *NGN2* transduction protocol (Fig. 1d, e). There were subtle changes in gene expression, with the most divergent genes shown in the bottom panel of Fig. 1d. Overall, immunostaining, RNA sequencing of pooled cultures, and single-cell Fluidigm analyses revealed consistent and comparable expression of mature excitatory neuronal markers in iNs regardless of *DISC1* genotype. *NGN2* transduced cultures demonstrated spontaneous action potentials by DIV 8 (Supp Fig. 2a, b). Spontaneous activity increased over differentiation time, but was not significantly different across the 4 genotypes studied (Supp Fig. 2c). These data show that two distinct *DISC1* mutations do not dramatically alter cell fate or activity in *NGN2*-transduced mature excitatory neurons.

### *DISC1* mutations similarly decrease DISC1 protein levels but do not globally dysregulate presynaptic protein expression

**Fig. 2:**
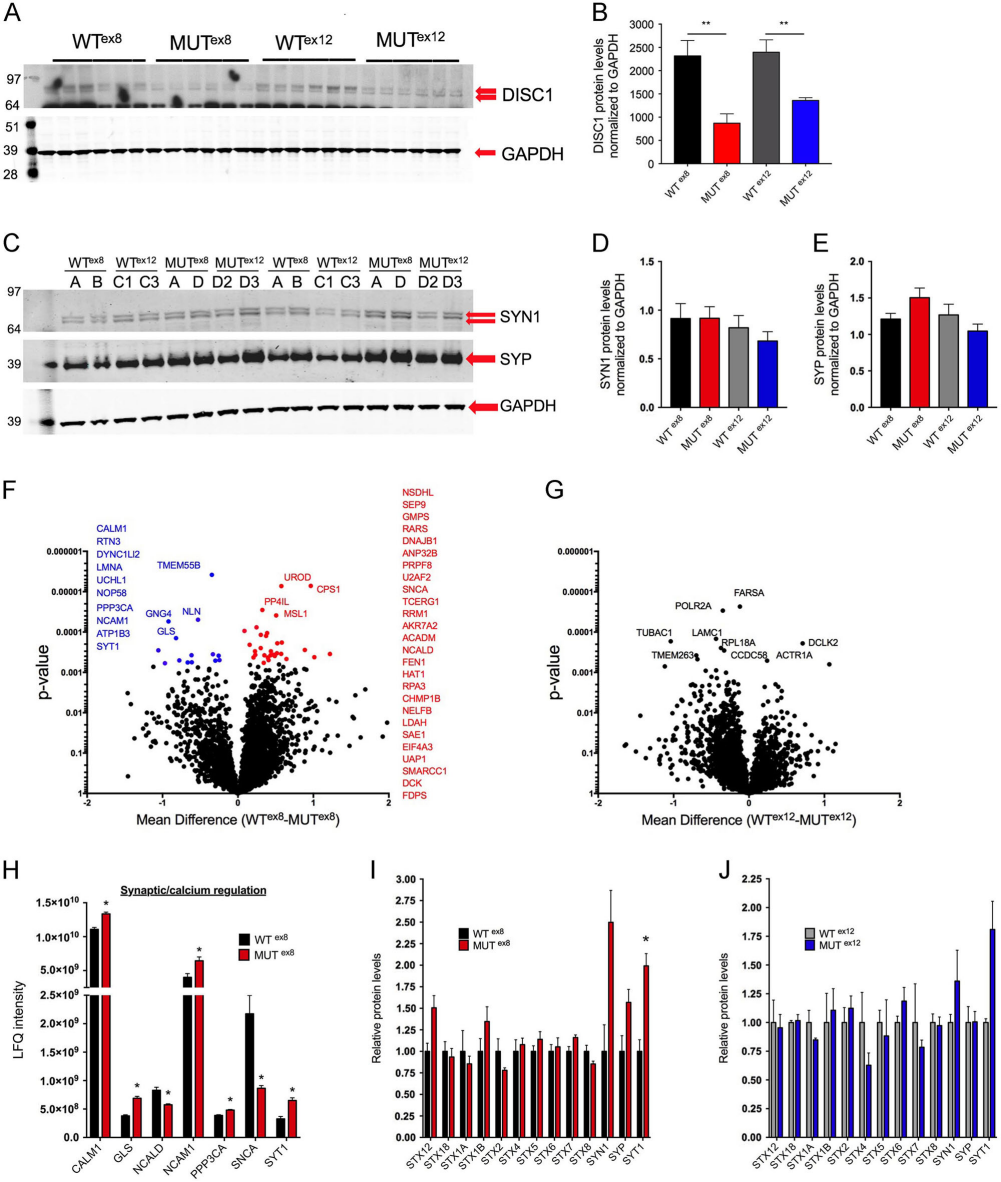
*DISC1* exon 8 and exon 12 mutations result in decreased DISC1 protein levels and altered levels of a subset of synaptic proteins. Day 21 iN cultures were lysed and used for western blot or proteomics as shown. **a** Representative DISC1 western blot. **b** Quantification of DISC1 expression by western blot, normalized to GAPDH. *n* = 6 for WT^ex8^, 6 for MUT^ex8^, 6 for WT^ex12^, 6 for MUT^ex12^. **c** Representative SYN1 and SYP western blot. **d**, **e** Quantification of SYN1 and SYP expression by western blot, normalized to GAPDH. *n* = 10 for WT^ex8^, 10 for MUT^ex8^, 10 for WT^ex12^, 10 for MUT^ex12^. Statistics **a**–**d**: Two-tailed Student’s *t*-test, **p < 0.01. **f**–**j** Day 21 iN lysates were used for proteomics by LC–MS/MS, *n* = 4 for each genotype. **f** Volcano plot of proteomic data of WT^ex8^ vs MUT^ex8^, with significantly upregulated genes colored blue and significantly downregulated genes colored red. **g** Volcano plot of proteomic data of WT^ex12^ vs MUT^ex12^, with no genes achieving statistical significance. **h** Differential expression of select proteins involved in neural adhesion, calcium signaling, and the synapse in MUT^ex8^ vs WT^ex8^ lysates. **i**, **j** Expression of presynaptic proteins by proteomics in WT^ex8^/MUT^ex8^**i** and WT^ex12^/MUT^ex12^**j**. Statistics **f**–**j**: Paired *t*-test with two-stage linear step-up procedure of Benjamini, Krieger, and Yejutieli, with false discovery rate *Q* = 5%. See also Supp. Table 2

The two *DISC1* mutations described each induce frameshift mutations at different locations in the coding sequence of the *DISC1* gene. However, the proposed mechanisms of these mutations differ. The exon 8 mutation has been shown to decrease DISC1 protein expression in iPSC-derived human neurons via non-sense-medicated decay of the frameshift mutation RNA^11^. Based on overexpression studies in HEK293 cells, the exon 12 mutation has been proposed to generate a mutant protein that aggregates with wild-type protein, depleting soluble DISC1^12^. Western blot of iN lysates showed similar decreases in protein levels to approximately half wild-type levels with both exon 8 and exon 12 mutation (Fig. 2a, b). This suggests that, regardless of mechanism, both mutations result in haploinsufficiency with a decrease in wild-type DISC1 protein expression. Prior studies in embryoid aggregate-derived neurons showed either no change in presynaptic protein expression (MUT^ex8^)^11^, or increased expression of presynaptic proteins (MUT^ex12^)^12^. Using the *NGN2* induction protocol in parallel, neither exon 8 nor exon 12 *DISC1* mutation significantly altered SYP or SYN1 expression (Fig. 2c, d).

Unbiased analyses of proteomic data comparing all *DISC1* mutant lines to all wild-type lines did not result in any changes in protein expression that were significant after correction for multiple comparisons. This was largely due to variability in protein expression, especially in the WT^ex12^ and MUT^ex12^ samples. As these 4 lines are familial but not isogenic, there may be increased variability both within and across genotypes, reducing the power to detect significant differences in protein expression. Separate paired analyses of WT^ex8^/MUT^ex8^ and WT^ex12^/MUT^ex12^ proteomic data confirmed this, as WT^ex8^/MUT^ex8^ analysis revealed multiple significant changes in protein expression (Fig. 2f), whereas WT^ex12^/MUT^ex12^ data showed no significant changes in expression (Fig. 2g). Altered protein expression with *DISC1* exon 8 mutation included dysregulation of proteins involved in synaptic and calcium regulation (Fig. 2h), cholesterol and lysosomal biology, chromatin regulation and DNA synthesis, RNA regulation, and the stress response (Supp Fig. 3). Proteomic analyses of both genotypes showed overall unchanged expression of many presynaptic proteins (Fig. 2i, j). Interestingly, however, and in line with the previous study of MUT^ex^^12^ neurons^12^, we observed a trend for increased expression of a subset of presynaptic proteins, with SYT1 being the only protein in this category that was significantly increased in MUT^ex8^ lysates (Fig. 2i, j).

### RNA sequencing shows altered expression of *UNC5D*, *PCDHA6, DPP10*, and *ZNF506* with both *DISC1* models

**Fig. 3:**
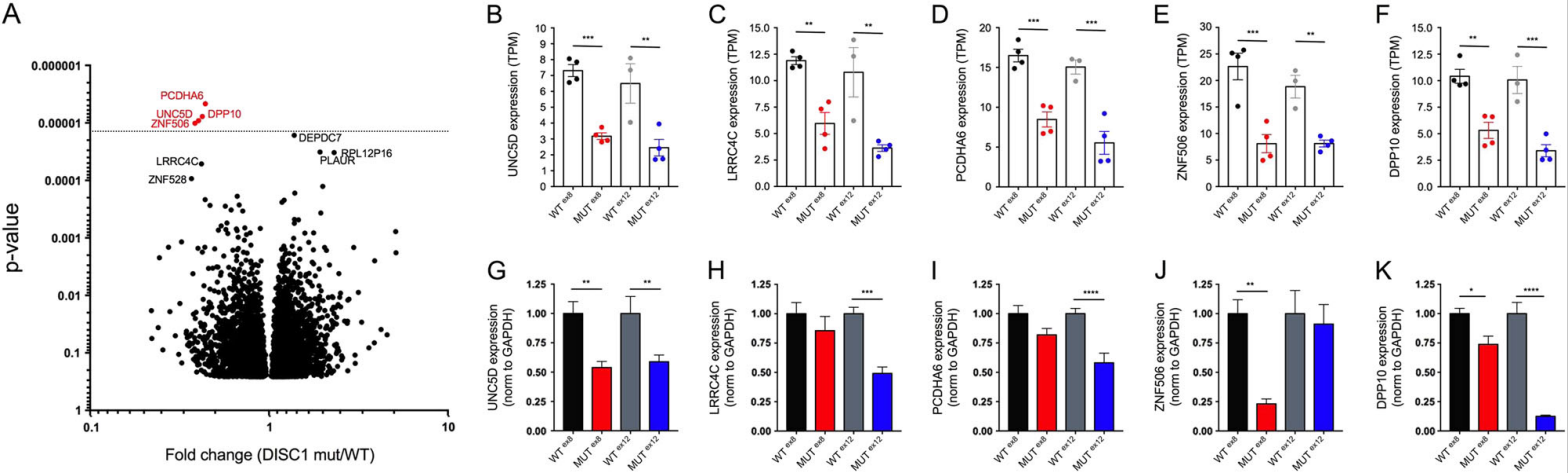
DISC1 exon 8 and exon 12 mutations converge on differential expression of a limited number of genes by RNA sequencing. **a**–**f** RNA was collected from day 21 iNs and used for RNA sequencing. *n* = 4 for WT^ex8^, 4 for MUT^ex8^, 3 for WT^ex12^, 4 for MUT^ex12^. **a** Volcano plot of RNA sequencing data of all wild type (WT^ex8^ and WT^ex12^) vs all mutant (MUT^ex8^ and MUT^ex12^). Statistics: two-stage linear step-up procedure of Benjamini, Krieger, and Yejutieli, with *Q* = 5%. Dotted line shows threshold of significance for *Q* = 5%. **b**–**f** RNAseq data for individual genes as shown, by genotype. **g**–**k** Day 21 iN culture RNA was collected and used for qRT-PCR for select genes as shown, normalized to GAPDH. *n* = 12 for WT^ex8^, 12 for MUT^ex8^, 12 for WT^ex12^, 12 for MUT^ex12^, from three independent differentiations. See also Supp. Table 3

As proteomic analyses are limited by peptide detection of only the most abundant proteins, with peptide detection representing ~4000–5000 proteins, we next performed RNA sequencing of day 21 iNs to assess gene expression changes at a whole-transcriptome level. Although there were similar challenges of variability as in proteomic data, analysis of all mutant (MUT^ex8-ex12^) vs all wild-type (WT^ex8-ex12^) RNA sequencing data showed decreased expression of 4 genes after genome-wide correction for multiple comparisons: *UNC5D*, *PCDHA6*, *DPP10*, and *ZNF506* (Fig. 3a). Representation of data across all 4 genotypes showed similar decreases in expression with MUT^ex8^ and MUT^ex12^ for each of these genes (Fig. 3b, d–f). As *UNC5D* encodes a netrin receptor, it was interesting that expression of *LRRC4C* (a member of the netrin family and a binding partner for netrin G1) also was decreased though did not meet statistical significance after multiple comparison correction (Fig. 3c). Of note, none of these gene products were detected in proteomic analysis.

RNA sequencing results were validated in separate RNA samples using qRT-PCR on three independent differentiations, which confirmed decreased expression of *UNC5D* and *DPP10* in MUT^ex8^ and MUT^ex12^ samples (Fig. 3g, k). The remaining genes showed significantly decreased expression in only one mutant genotype by qRT-PCR (Fig. 3h–j). Although variability of expression across lines and genotypes limited the discovery of significant gene expression changes, the few identified here were robust enough to be significant at a whole-transcriptome level despite this variability. Furthermore, decreased *UNC5D* and *DPP10* expression was validated in MUT^ex8^ and MUT^ex12^ genotypes in independent samples by qRT-PCR.

### Independent *DISC1* mutations converge on reduced neurite outgrowth via decreased *UNC5D* expression

**Fig. 4:**
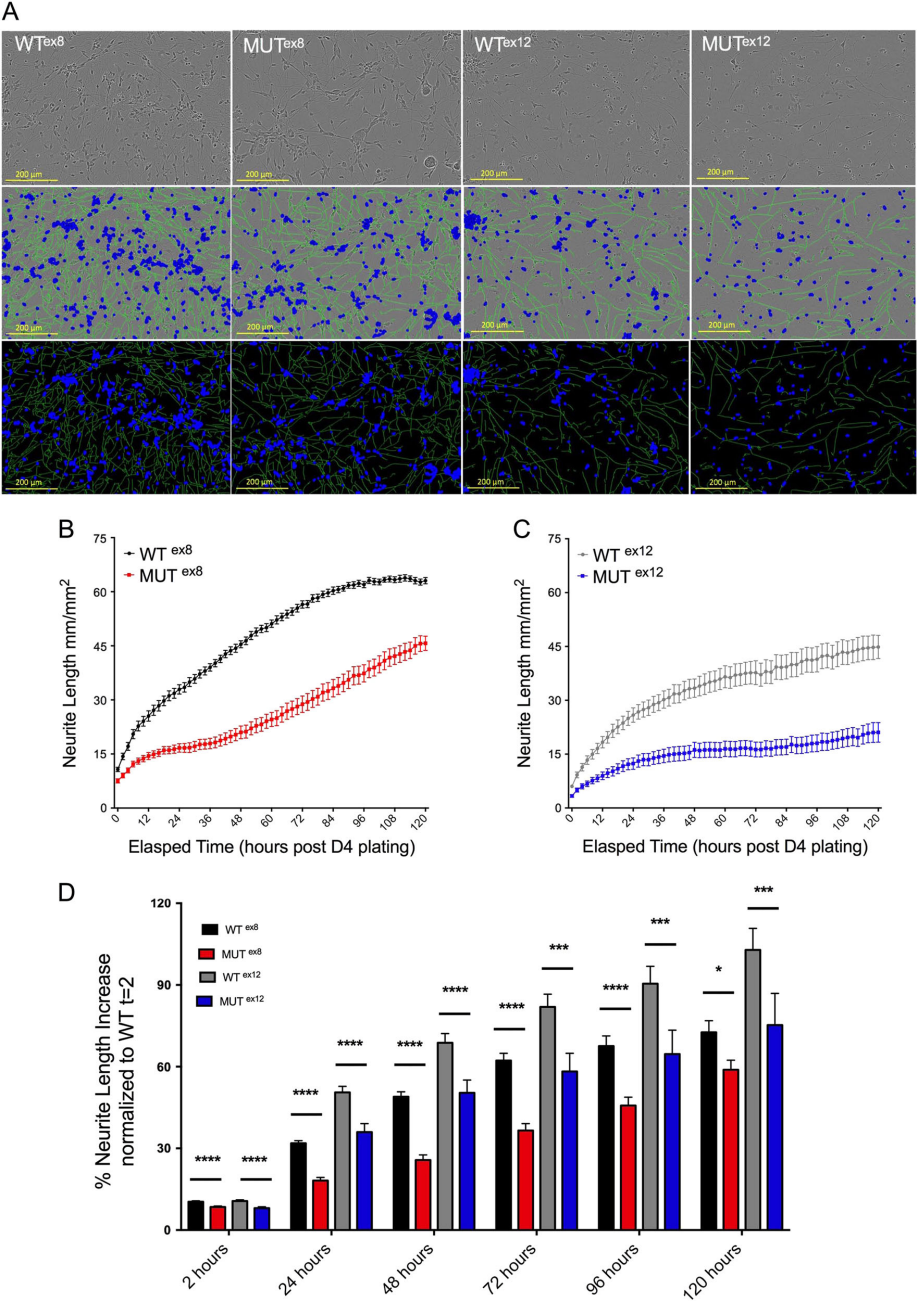
DISC1 mutant neurons share a common phenotype of impaired neurite outgrowth. Day 4 iNs were dissociated and plated for longitudinal IncuCyte imaging and analysis of neurite length. **a** Example images from the IncuCyte are shown from day 9 cultures (120 h post D4 plating). Top row = brightfield, middle row = overlay of cell body mask in blue and neurite mask in green on brightfield, bottom row = cell body mask in blue and neurite mask in green. Scale bars = 200 μm. **b**, **c** Example longitudinal measurements of neurite length are shown from one experiment for WT^ex8^ vs MUT^ex8^**b** and WT^ex12^ vs MUT^ex12^**c**. **d** Neurite length measurements from 2 to 120 h for all 4 genotypes are shown, with WT^ex8^ and MUT^ex8^ data normalized to WT^ex8^*t* = 2 h and WT^ex12^ and MUT^ex12^ data normalized to WT^ex12^*t* = 2 h. *n* = 6 for each group, three independent differentiations. Statistics: Holm-Sidak method with multiple comparison correction, FDR = 5%. *q < 0.05, ***q < 0.001, ***q < 0.0001

UNC5D is a co-receptor for netrin with the netrin receptor DCC, and modulates DCC signaling. UNC5D is expressed in the subventricular zone (SVZ) and upper layer neurons, and has been found to regulate the transition to radial migration^39–43^. The interaction of netrins with netrin receptors allow for chemotropic guidance of migrating axons and cells in the developing brain^44^. *DISC1* has independently been implicated in neurite outgrowth via its interacting proteins as well as in models of *DISC1* mutation^12,45–51^. In order to study the effects of *DISC1* mutation on neurite outgrowth in postmitotic neurons, we used the IncuCyte live cell imaging system to morphologically assay differentiating iNs from all genotypes starting at day 4, allowing for automated measurements of neurite characteristics over time. These studies showed that both exon 8 and exon 12 *DISC1* mutations resulted in significant decreases in neurite length relative to wild-type controls, which persisted up to 120 h in vitro (DIV 9, Fig. 4a–d).

**Fig. 5:**
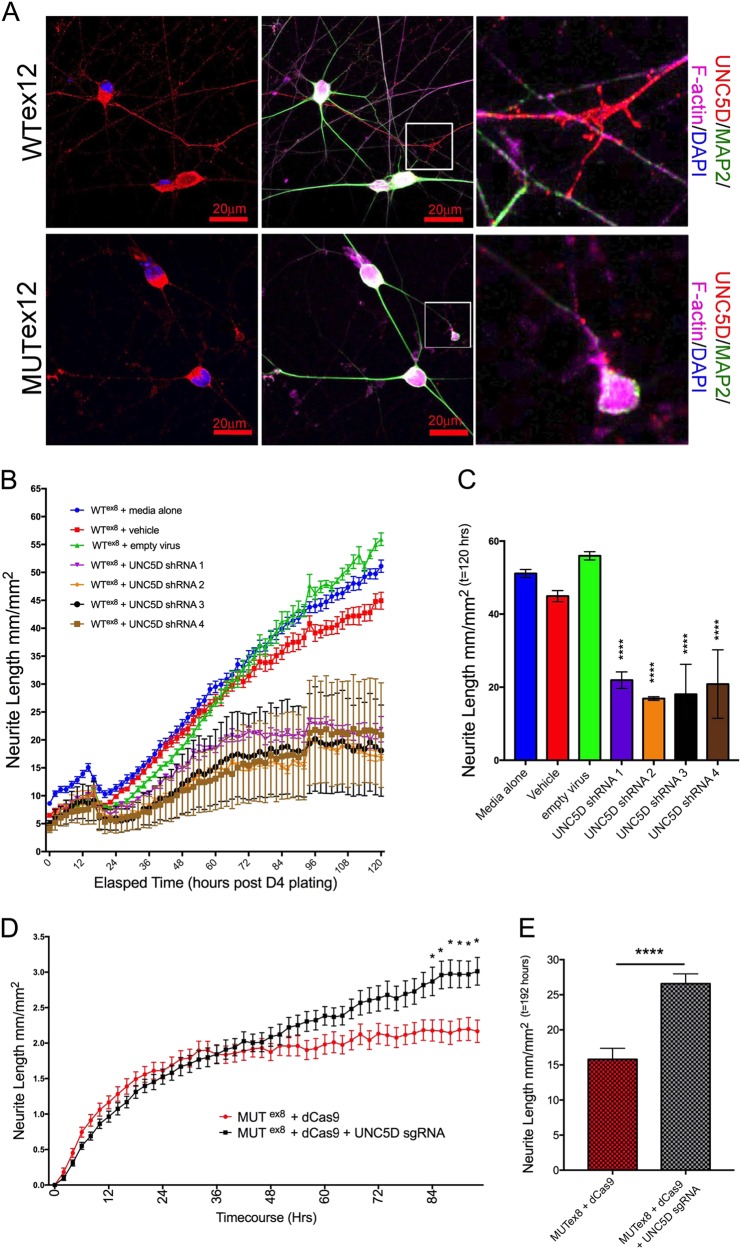
Decreased neurite outgrowth with *DISC1* mutation can be mimicked with *UNC5D* downregulation and rescued with *UNC5D* upregulation. **a** Day 9 iNs were immunostained for UNC5D, MAP2, and F-actin as shown, revealing UNC5D expression in neurites, dendritic spines, and growth cones. Scale bar = 20 μm. **b**–**c** *UNC5D* shRNAs were transduced into day 4 iNs. WT^ex8^ day 4 iNs were dissociated and plated for IncuCyte neurite length analysis with media alone (vehicle), viral transduction vehicle (empty), or else transduced with empty virus or 4 shRNAs targeting different sequences within UNC5D. Longitudinal neurite length analysis is shown. **d**, **e** MUT^ex8^ day 4 iNs were dissociated and transfected with either dCas9-VP64 + RFP (dCas9) or dCas9-VP64 + UNC5D sgRNA #2 + RFP (dCas9 + UNC5D sgRNA). RFP-positive neurite length was analyzed longitudinally with IncuCyte. **e** Neurite length analysis at 192 h after plating (DIV 9) shows significantly increased neurite length with transient transactivation of endogenous *UNC5D* expression with gRNA #2. *n* = 25 for each group. Statistics: one-way ANOVA, *p < 0.05, **p < 0.01, ***p < 0.001, ***p < 0.0001

In order to investigate the effects of altered *UNC5D* expression on neurite outgrowth, we manipulated *UNC5D* expression and measured neurite outgrowth. In order to first visualize whether UNC5D was expressed at the protein level, we performed immunostaining in day 21 iNs. Immunostaining for UNC5D showed localization to neurites, dendritic spines, and growth cones (example images shown in Fig. 5a). Knockdown of *UNC5D* expression with four independent shRNAs resulted in a decrease in *UNC5D* expression in iNs (expressed as % expression relative to controls, mean ± SD: shRNA #1–66.5 ± 11.4, shRNA#2–57.5 ± 7.4, shRNA#3: 77.2 ± 10.3, shRNA#4: 77.1 ± 9.3). Viral transduction of day 4 iNs with each of these shRNAs impaired neurite outgrowth in wild-type iNs (Fig. 5b, c), mimicking the effect of *DISC1* exon 8 and exon 12 mutations. We then upregulated endogenous *UNC5D* expression using a catalytically inactive Cas9 fused to C-terminal VP64 acidic transactivation domain^36,37^ targeted to the *UNC5D* promoter sequence. Transfection of these plasmids dramatically increased UNC5D expression in HEK293T cells (Supp Fig. 4A). Although, the transfection efficiency of iNs was low, making increased *UNC5D* expression difficult to assess in pooled cultures, co-transfection of UNC5D-targeted dCas9-VP64 with RFP allowed for neurite length analysis of only RFP-positive, transfected neurites (Supp Fig. 4B). Transient transactivation of *UNC5D* expression in MUT^ex8^ iNs resulted in increased RFP-positive neurite length relative to control by 84 h after transfection, rescuing the *DISC1* mutant phenotype (Fig. 5d, e). These data suggest that two independent *DISC1* mutations result in impaired neurite outgrowth in vitro that is mediated via decreased *UNC5D* expression.

## Discussion

The advent of “induced neuron” differentiation methods, which accelerate differentiation by overexpressing master regulators of cell fate, allows for detailed study of fate-restricted homogenous populations of mature neurons. In the present study, we use one such method to study the effects of two distinct *DISC1* mutations in mature excitatory neurons. *NGN2* transduction results in homogenous and mature neuronal cultures, by pooled and single cell gene expression analyses. We find that independent *DISC1* mutations decrease levels of detergent-soluble DISC1 protein, consistent with haploinsufficiency. In this cellular model, these mutations do not dramatically dysregulate presynaptic protein expression or spontaneous neural electrophysiological activity. Both *DISC1* mutations result in significant decreases in *UNC5D* and *DPP10* expression by unbiased RNA sequencing, confirmed by qRT-PCR. Examination of neurite outgrowth in culture revealed an early and persistent decrease in neurite length with *DISC1* exon 8 and exon 12 mutations, which was phenocopied by *UNC5D* knockdown and rescued by *UNC5D* upregulation.

These neurite outgrowth findings contrast the prior study of MUT^ex12^ neurons, wherein transient increases in neurite length were observed with DISC1 mutation^12^. The protein expression findings also differ from the prior study, which found dramatic upregulation of the presynaptic protein apparatus and decreased spontaneous activity in iPSC-derived neurons^12^. These differences may be a result of the distinct differentiation protocols used—the prior study utilized an embryoid-aggregate method, which recapitulated neurodevelopment, whereas this study bypasses early neurodevelopment via rapid induction of *NGN2*. Both differentiation protocols have benefits for understanding the neurobiology of DISC1 mutations; however, in order to study convergent effects across two separate mutations, utilizing a protocol that generated a very homogeneous population of neurons ensured less variability across iPSC lines. By using one differentiation method vs another, different results in phenotypes may be uncovered due to the specific population of neurons being studied.

The proteome and transcriptome analyses described here were limited by variability across iPSC lines. This was especially true with the WT^ex12^/MUT^ex12^ lines, which are intra-familial but not isogenic. Conversely, the isogenic WT^ex8^/MUT^ex8^ lines demonstrated increased power to detect significant differences in gene and protein expression, perhaps due to reduction of other genomic variability or else to the relative potency of the mutations. This demonstrates the importance of using isogenic controls in order to strengthen analyses of disease-associated phenotypes. However, identification of gene expression changes and impaired neurite outgrowth even with non-isogenic genomic variability in WT^ex12^/MUT^ex12^ lines demonstrates the robustness of these phenotypes.

While proteomic analysis did not show dramatic dysregulation of synaptic proteins, analysis of WT/MUT^ex8^ lines did reveal altered expression of proteins implicated in neuronal physiology, including the neuronal migration protein CALM1^52^, glutamate generating enzyme GLS, Ca^++^-sensing protein NCALD^53^, cell adhesion protein NCAM (implicated in neurite outgrowth, neuronal migration, and synaptic plasticity^54^), synaptic vesicle recycling protein PPP3Ca (calcineurin A; implicated in neurodevelopmental disorders^55,56^), alpha-synuclein (SNCA), and SYT1 (a Ca^++^ sensor critical for fast neurotransmitter release^57^). These expression perturbations suggest that there may be a subtle dysregulation of synaptic biology in mature glutamatergic neurons with *DISC1* disruption, which was only detectable in comparison of isogenic WT/MUT^ex8^ neurons.

We compared the RNAseq data in the current study to previously published RNAseq analyses in neurons with *DISC1* disruption. We found that *UNC5D*, related gene *UNC5C*, and *LRRC4C* were downregulated with DISC1 exon 2 homozygous mutation in day 50 embryoid aggregate-derived neurons; *ZNF506* was downregulated in MUT^ex8^ day 50 embryoid aggregate-derived neurons; and *LRRC4C* and *ZNF506* were downregulated in MUT^ex8^ day 17 embryoid aggregate-derived NPCs^11^. The *UNC5D*-related genes *UNC5B* and *UNC5C* were downregulated in MUT^ex12^ day 28 embryoid aggregate-derived neurons^12^. Despite differences in differentiation protocol and neural cell fate, overlapping gene expression changes of the current study with previous analyses strengthen the association of the observed gene expression changes with *DISC1* mutation.

Both *DISC1* mutations studied here resulted in a reproducible decrease in *UNC5D* expression, which was mechanistically linked to decreased neurite outgrowth in *DISC1*-disrupted neurons. The UNC5 proteins are netrin co-receptors that have been described to interact with DCC to mediate repulsive cues^58^. UNC5D is expressed in rodents in multipolar migrating cells of the SVZ and intermediate zone (IZ)^39,40^, as well as layer four cortical neurons^40,43,59,60^. Migrating cortical projection neurons initially have a bipolar morphology in the ventricular zone (VZ), then transition to a multipolar morphology in the SVZ/IZ where cells extend and retract processes dynamically^61^, and eventually extend an axon prior to resuming radial migration, transforming back to a bipolar cell morphology and entering the cortical plate^41^. We previously had shown that DISC1 mutation alters FOXG1 levels^11^, a telencephalic transcription factor that represses UNC5D expression^39^. Dynamic FOXG1 expression results in upregulation of UNC5D (required for initiation of multipolar migration), followed by downregulation of UNC5D (required for multipolar-to-bipolar transition and subsequent cortical plate entry)^39^. UNC5D has also been shown to interact with Netrin-4 to regulate cell survival and interacts with FLRT2 to delay radial migration into the cortical plate via repulsive cue signaling^60,62,63^. Decreased expression of *UNC5D* and consequent impaired neurite outgrowth with disease-associated *DISC1* mutations thus suggests a possible perturbation of axonal guidance and dysregulation of chemotropic factor-guided radial migration. Interestingly, the *UNC5D* locus has been associated with cortical brain volume in the Framingham study^64^, which could implicate this gene in the decreased cortical thickness seen with *DISC1* polymorphisms and mutation^6,65,66^.

The netrin family gene *LRRC4C*, which was decreased in MUT^ex8^ and MUT^ex12^ by RNAseq (but did not meet threshold for whole-transcriptome significance) and in MUT^ex12^ by qRT-PCR, has been associated with neurodevelopmental disorders in genetic studies^67^. LRRC4C (also referred to as NGL-1) is a netrin-G1 receptor that promotes axonal outgrowth^68^, and the NGL family is involved in excitatory synapse formation^69^. LRRC4C is also phosphorylated by autism spectrum disorder vulnerability gene CDKL5, which modulates the interaction of LRRC4C with PSD-95^70^. Furthermore, Netrin-G1 (NTNG1) disruptions or polymorphisms have been associated with atypical Rett syndrome, intellectual disability, autism spectrum disorder, and schizophrenia^71–74^. Decreased expression of both *LRRC4C* and *UNC5D* further strengthens the association of *DISC1* disruption with perturbation of cell adhesion and chemotropic guidance molecule-mediated axonal guidance and cell migration. These previously described roles for netrin signaling do not preclude additional roles for UNC5D and LRRC4C, but provide clues to their function in the mature brain.

The other genes identified by RNAseq to be altered with *DISC1* exon 8 and exon 12 mutation are *DPP10, PCDHA6*, and *ZNF506*. DPP10 modulates the activity of Kv4.2 potassium channels, and has been implicated in mental illness and neurodegenerative disease models^75–77^ Decreased *DPP10* expression suggests a possible synaptic phenotype not identified in the current study, which could be further investigated with detailed electrophysiological analyses. *PCDHA6* is a member of the alpha protocadherins, a family of Ca^++^-dependent cell adhesion molecules^78^ that have been linked to bipolar disorder and schizophrenia^79^. ZNF506 is a zinc-finger protein without known function.

DISC1 has been implicated in neuronal migration and neurite outgrowth in several studies (reviewed here^1^). *DISC1* knockdown reduces migration of cultured mouse NPCs^80^, in vivo rodent cortical neurons^81–84^ and interneurons^48^. *DISC1* polymorphisms have also been linked to impaired migration^85^, and DISC1 phosphorylation has been liked to a switch from NPC proliferation to migration^86^. In rodents, *DISC1* polymorphisms or mutation have been linked to decreased neurite length in vivo^47,87^ and in primary neuronal cultures^88^. DISC1 has been linked to neurite outgrowth in immortalized rodent cell cultures, with proposed mechanisms of interactions with NDEL1, FEZ1, DBZ, or dysbindin^46,50,81,91–93^. Study of the *DISC1* exon 8 mutation in a three-dimensional cerebral organoid system revealed a marked WNT-dependent morphologic alteration^94^, which could reflect an alteration in migration related to the decrease in *UNC5D* expression observed here. Indeed, UNC5D RNA levels were found to be reduced in DISC1 ex8 organoids (see Supp Fig. 5).

Although, the causal link between *DISC1* mutation and major mental illness remains controversial, the relationship of DISC1 to neurodevelopment has been well-established^1^. The current study shows that multiple mutations previously linked to mental illness result in shared gene expression changes and decreased neurite outgrowth related to decreased *UNC5D* expression. Further study of multiple neuropsychiatric disease-associated mutations in parallel will allow for identification of convergent phenotypes and elucidate shared mechanisms of neurodevelopmental disorder pathophysiology.

**Table. 1:** Primers

Gene	NCB1 gene ID	Primer	Sequence
*GAPDH*	2597	Forward	GGGAGCCAAAAGGGTCATCA
		Reverse	TGGTTCACACCCATGACGAA
*UNC5D*	137970	Forward	CTTTAGGAAGCGATCGTGGAG
		Reverse	GAAAGGGTCGGCGATGAG
*DPP10*	57628	Forward	AGACTTGCCTTCCTGATGATAAA
		Reverse	CACTTGACCTGCCTTAGGATAC
*PCDHA6*	56142	Forward	GGAAAGCAATGTCTGCTCCTC
		Reverse	CCTCCTCGGGTACGGAGTAG
*ZNF506*	440515	Forward	CTGTCCTGTTCTGTTCCATTCT
		Reverse	CCCTCTTAAGGGCTTACAACTC
*LRRC4C*	57689	Forward	GGTGATTTGTGTTCGGAAAAACC
		Reverse	CGGGATGGTAGTAAGACGATTG

**Table. 2:** Antibodies

Antigen	Host	Application	Dilution	Vendor	Catalog #
MAP2	Chicken	ICC, WB	1/2000	Abcam	ab5392
BRN2	Rabbit	ICC	1/300	Abcam	ab137469
CUX2	Rabbit	ICC	1/200	Abcam	ab130395
VGLUT1	Rabbit	ICC	1/300	Synaptic systems	135303
VGLUT2	Mouse	ICC	1/1000	Abcam	ab79157
Tau (MAPT)	Rabbit	ICC, WB	1/200	Dako	A0024
SYP	Rabbit	ICC, WB	1/200, 1/1000	Abcam	ab14692
DISC1 (3G10)	Mouse	WB	1/1000	TYP lab^12^	
GAPDH	Mouse	WB	1/2000	Millipore	MAB374
SYN I	Rabbit	WB	1/200	Millipore	574777
UNC5D	Mouse	ICC	1/500	Abcam	ab54430
TUJ1	Mouse	ICC	1/1000	Millipore	MAB1637
NeuN	Mouse	ICC	1/1000	Millipore	MAB377

## Electronic supplementary material

Supplementary Information

Supplementary Figure 1

Supplementary Figure 2

Supplementary Figure 3

Supplementary Figure 4

Supplementary Figure 5

Supplementary Tables
