# Supplementary material for: Convergence of independent DISC1 mutations on impaired neurite growth via decreased UNC5D expression
Source: Transl Psychiatry. 2018 Nov 8;8:245. doi: 10.1038/s41398-018-0281-9 (PMC6224395; doi:10.1038/s41398-018-0281-9)
Supplement: Supplementary file 1 — Supplementary Information [file 41398_2018_281_MOESM1_ESM.docx]

**Supplemental Figure legends**

**Supplemental Figure 1. *NGN2* transduction results in decreased expression of progenitor markers and increased expression of mature neuronal markers compared to embryoid aggregate differentiation.** WT^ex8^ cells were differentiated via an embryoid aggregate (or embyroid body, EB) method or via NGN2 transduction to generate induced neurons (iN). RNA was harvested from wild-type embryoid aggregate day 40 and induced neuron (iN) day 28 cultures and used for the NanoString assay to compare gene expression. Expression was normalized across all genes, and then to average EB data. (A) Markers of neural progenitor fates. (B) Markers of general neural progenitor and neuronal fates. (C) Markers of neuronal maturity. Data derived from 3-6 independent differentiations. Statistics: Holm-Sidak method, * p<0.05, ** p<0.01, *** p< 0.001, *** p<0.0001.

**Supplemental Figure 2. *NGN2* transduction generates spontaneously active neurons.** Day 4 iN cells were dissociated and plated with human astrocytes on microelectrode arrays. (A) Example raster plots of day 29 iN cultures. Each row represents one electrode, 8 representative electrodes from 1 recording are shown. (B) A representative day 24 single unit waveform is shown. (C) Mean firing rate (MFR) from MEAs using WT^ex8^, MUT^ex8^, WT^ex12^, or MUT^ex12^ lines, binned by different time points of differentiation as shown. Y axis represents MFR per node of a 96 well MEA plate. Data were filtered to remove nodes with a MFR <0.0005 Hz. N = 4 for all genotypes through day 45, then: day 46-55 N = 4 WT/MUT^ex8^ and N = 2 WT/MUT^ex12^; day 56-71 N = 2 for all genotypes.

**Supplemental Figure 3. Differential expression of select proteins by LC-MS/MS proteomics with *DISC1* exon 8 mutation.** Day 21 iN cultures were lysed and used for proteomics by LC-MS/MS, n=4 for each genotype. Selected proteins are shown that were differentially expressed in MUT^ex8^ vs WT^ex8^ lysates, including proteins implicated in cholesterol/lysosomal biology (A), chromatin regulation/DNA synthesis (B), RNA regulation/proliferation (C), or the stress response (D). While not significant following multiple comparisons, the two most changed proteins when all WT and MUT data were combined were PTGFRN (E) and TARDBP (F).

**Supplemental Figure 4. Transfection of iNs with dCas9-VP64 + RFP, showing induction of endogenous UNC5D in HEKs and low transfection efficiency in iNs.**

(A) Endogenous *UNC5D* expression was upregulated by transfection with catalytically inactive Cas9 fused to VP64 transactivation domain (dCas9) with guide RNA targeted to the *UNC5D* promoter (UNC5D sgRNA). HEK293T cells were transfected with empty vector alone or dCas9-VP64 + UNC5D sgRNAs. RNA was harvested at 72 hours after transfection and used for qRT-PCR. sgRNAs #2 and #3 resulted in dramatic upregulation of *UNC5D* expression. n = 3 for each group. (B) MUT^ex8^ iNs were transfected at day 4 with dCas9-VP64 + RFP alone (left column) or dCas9-VP64 + UNC5D sgRNA + RFP (right column). Images shown were obtained at 192 hours after plating. Top row: overlay of RFP and brightfield images, showing low transfection efficiency. Middle row: RFP images alone. Bottom row: RFP images with IncuCyte neurite mask overlay in green. Scale bar = 200 μm.

**Supplemental Figure 5. DISC1 ex8 mutant organoids show a reduction in UNC5D at the RNA level.**

QPCR was performed for UNC5D on RNA derived from day 19 organoids (described in ^95^). Data were derived from three independent differentiations. Statistics: Mann-Whitney T-test, * p<0.05.

**Supplemental Methods**

**NanoString Assay**

A custom CodeSet designed by NanoString Technologies was utilized to analyze gene expression for 150 genes from each sample. Assays were performed according to the manufacturer’s instructions. Briefly, 200-1000 ng RNA was hybridized with capture and reporter probesets at 65°C for 12-30 hours. Hybridized samples were processed using an nCounter Prep Station and imaged using an nCounter Digital Analyzer (NanoString Technologies). Data were analyzed with nSolver Analysis Software (NanoString Technologies) and normalized to the total gene set.

**Microelectrode array recordings**

MEA recordings were performed as previously described^93^. Sterile 96-well MEA culture dishes (8 electrodes/well) were coated with poly-O-laminin (Sigma) and left to incubate at 37°C overnight. Wells were then washed 3 times with PBS before coating electrode area of well with Matrigel for 1 hr at 37°C. Induced neuron cultures were dissociated on day 4 of differentiation with Accutase + 10 uM ROCK Inhibitor + DNase I (5U/ml, NEB). Primary rodent astrocytes were cultured using DMEM (Gibco) and 10% FBS. Approximately 32000 neurons and 32000 rodent astrocytes were resuspended in D4 iN media supplemented with 2% FBS (omitting puromycin), in a 50 ul volume, and plated directly onto the electrode surface. Cells were incubated at 37°C for 2 hours to allow cells to adhere before flooding the wells with D4 iN media supplemented with 2% FBS. Cells were fed with NBM media with 2% FBS until D28, at which point they were fed BrainPhys (STEMCELL Technologies) until the end of the differentiation.

MEA recordings were performed using the Muse system (Axion Biosystems). Data were acquired using AxIS software (Axion Biosystems) at a sampling rate of 12.5 kHz, filtered using a 200-2500 Hz Butterworth band-pass filter. The detection threshold was set to ±5.5x SD baseline electrode noise. Raster plots were analyzed using NeuroExplorer (NEX Technologies). Waveform data were filtered and plotted using custom MATLAB scripts (The Mathworks).

**Supplemental Tables**

**Supplemental Table 1.** Single-cell qRT-PCR (Fluidigm) data from WT^ex8^ and MUT^ex8^ day 28 iNs after co-culture with mouse astrocytes.

**Supplemental Table 2.** Proteomics data from WT^ex8^, MUT^ex8^, WT^ex12^, and MUT^ex12^ pooled day 21 iNs.

**Supplemental Table 3.** RNA sequencing data from WT^ex8^, MUT^ex8^, WT^ex12^, and MUT^ex12^ pooled day 21 iNs.
