# Supplementary figures and images for: Convergence of independent DISC1 mutations on impaired neurite growth via decreased UNC5D expression
Source: Transl Psychiatry. 2018 Nov 8;8:245. doi: 10.1038/s41398-018-0281-9 (PMC6224395; doi:10.1038/s41398-018-0281-9)

Supplementary Figure 1

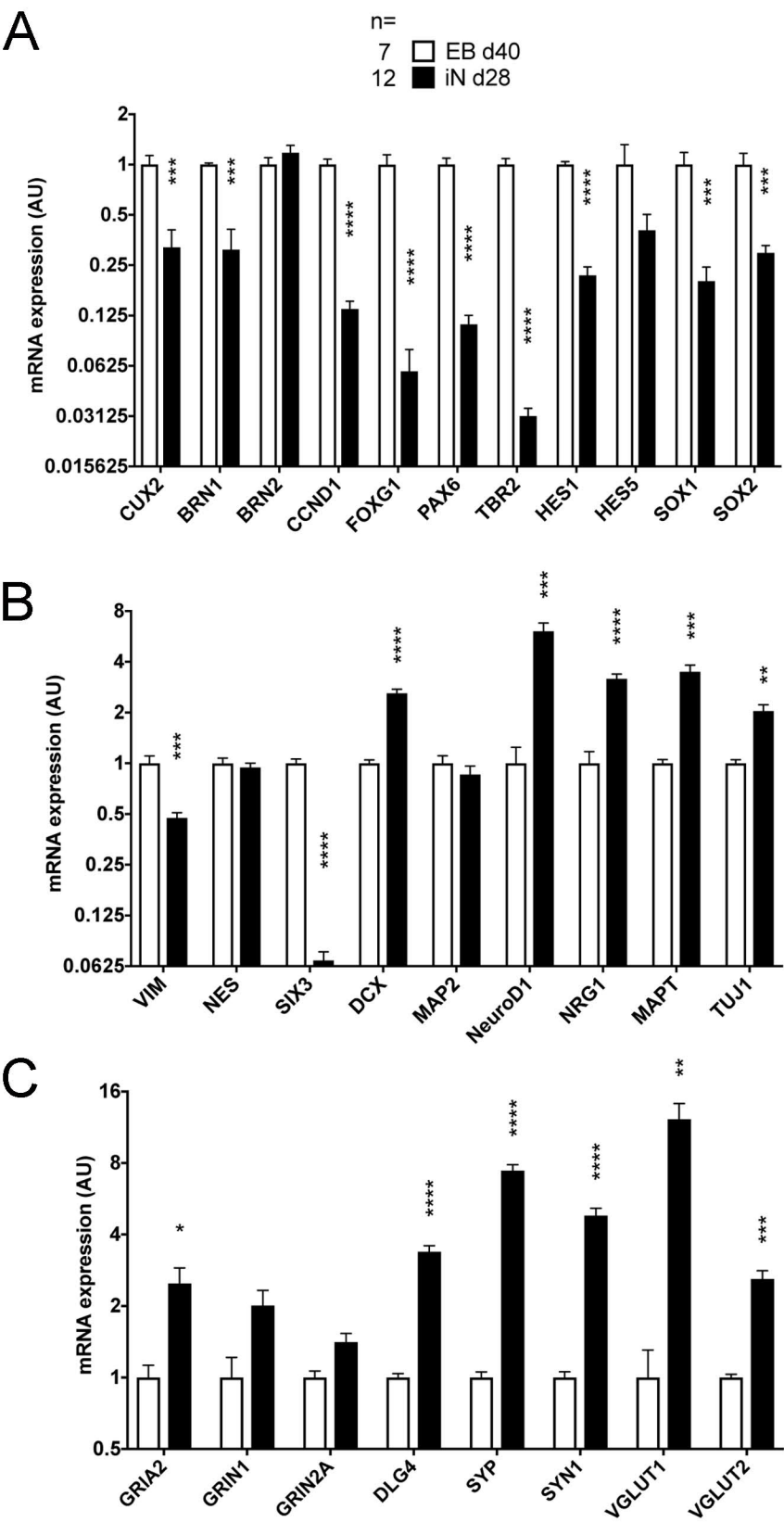

Supplement: Supplementary file 2 — Supplementary Figure 1 [file 41398_2018_281_MOESM2_ESM.pdf]

Supplementary Figure 2

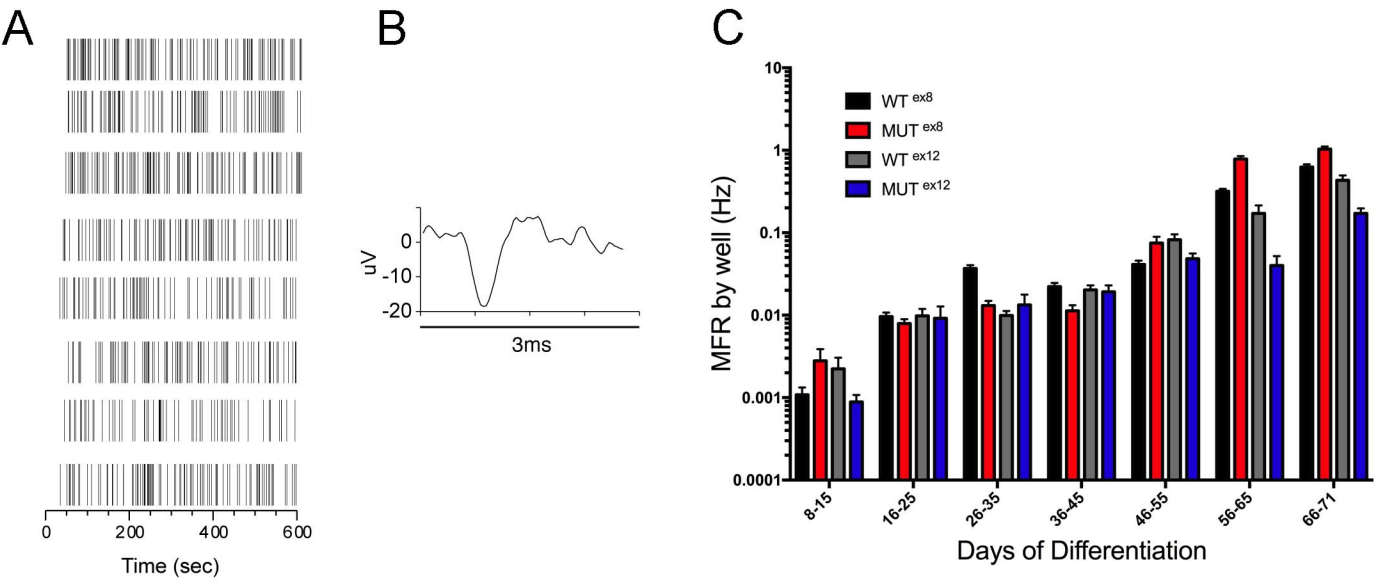

Supplement: Supplementary file 3 — Supplementary Figure 2 [file 41398_2018_281_MOESM3_ESM.pdf]

Supplementary Figure 3

A

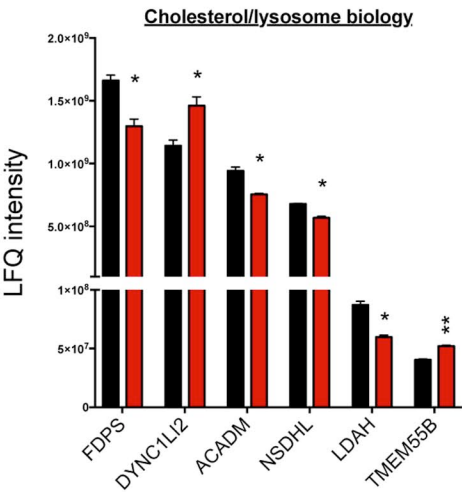

B

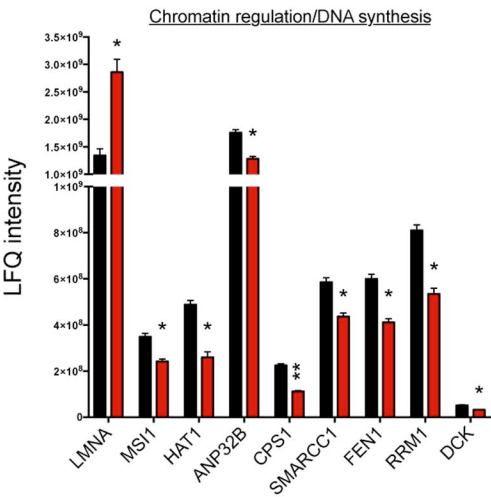

C

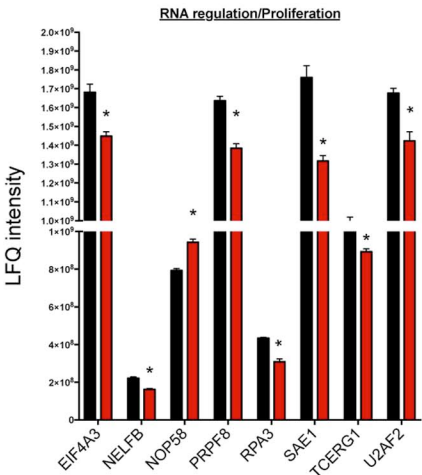

D

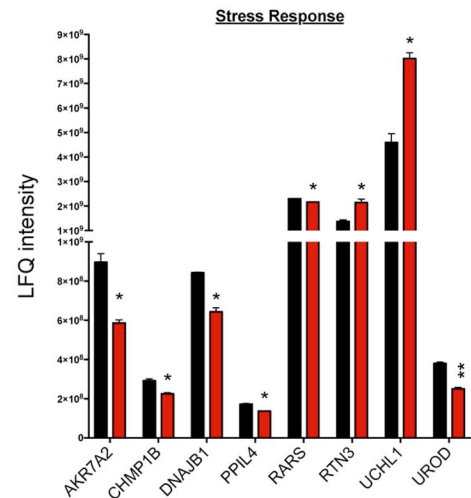

E

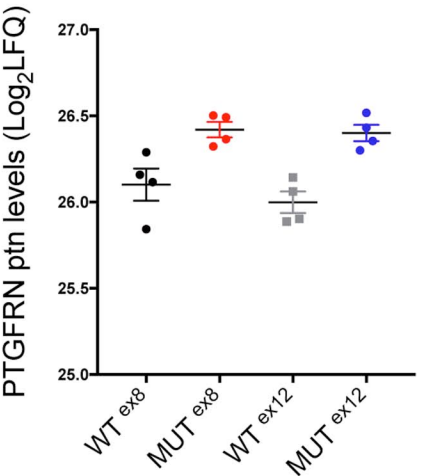

F

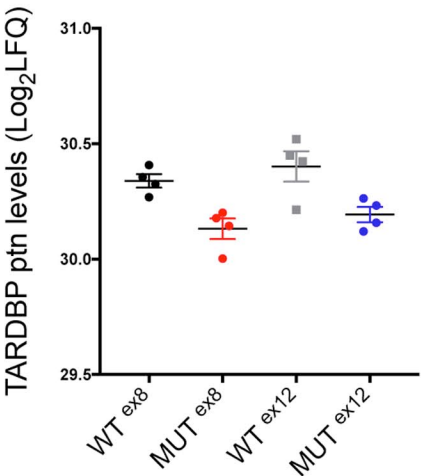

Supplement: Supplementary file 4 — Supplementary Figure 3 [file 41398_2018_281_MOESM4_ESM.pdf]

Supplmentary Figure 4

A

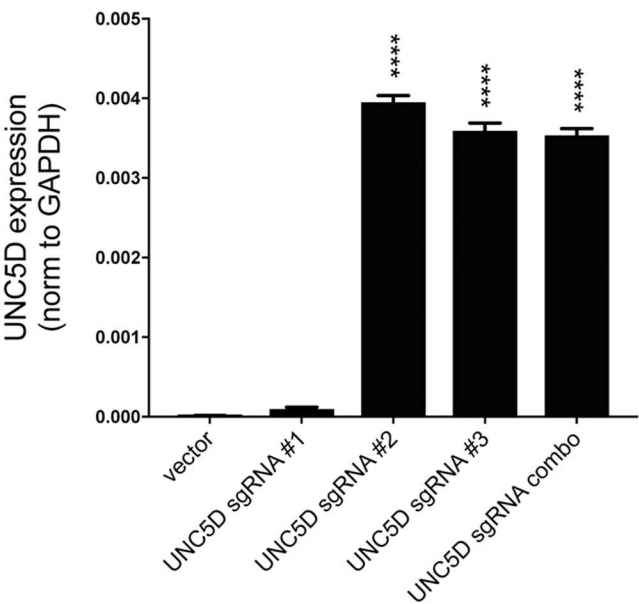

B

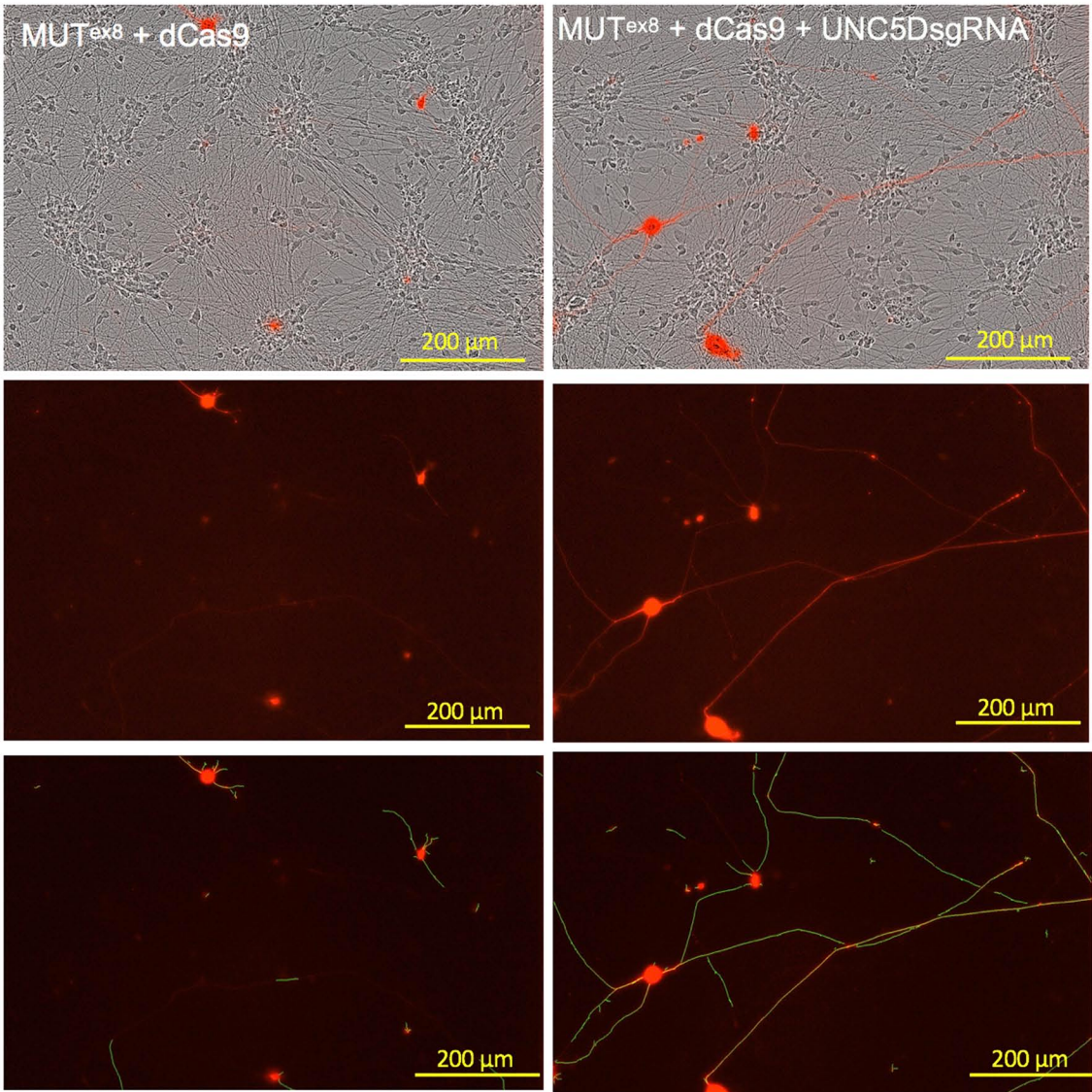

Supplement: Supplementary file 5 — Supplementary Figure 4 [file 41398_2018_281_MOESM5_ESM.pdf]

Supplementary Figure 5.

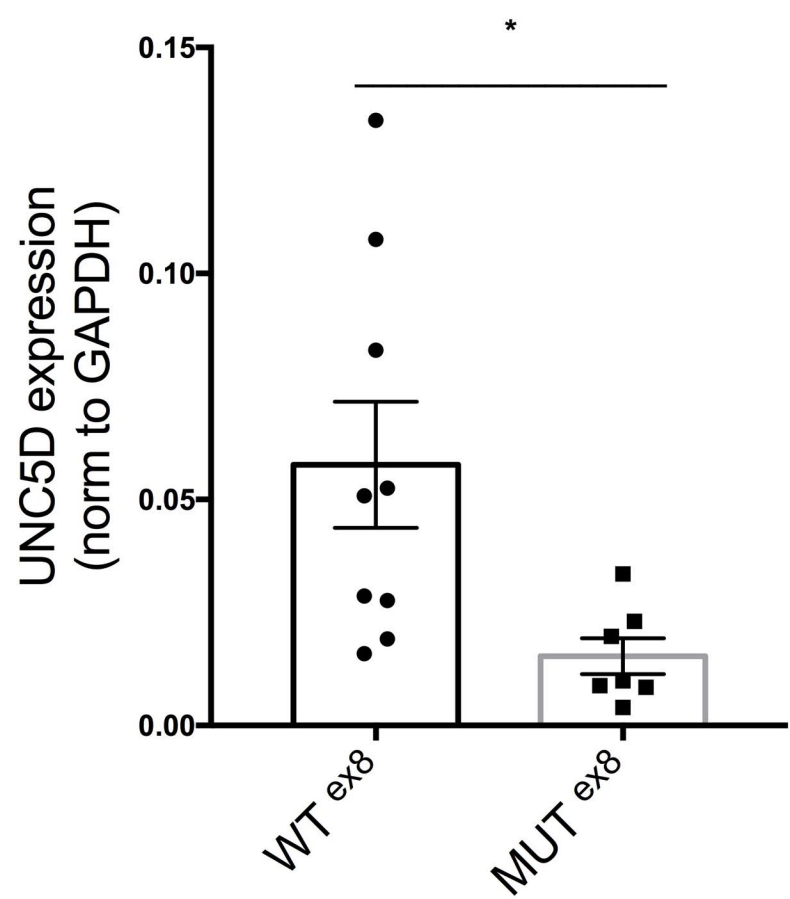

Supplement: Supplementary file 6 — Supplementary Figure 5 [file 41398_2018_281_MOESM6_ESM.pdf]
